# The Potential of Prebiotic and Probiotic Supplementation During Obese Pregnancy to Improve Maternal and Offspring’s Metabolic Health and Reduce Obesity Risk—A Narrative Review

**DOI:** 10.3389/fnut.2022.819882

**Published:** 2022-04-07

**Authors:** Eliane B. Wiedmer, Isabelle Herter-Aeberli

**Affiliations:** Laboratory of Human Nutrition, Department for Health Science and Technology, Institute of Food, Nutrition and Health, ETH Zürich, Zurich, Switzerland

**Keywords:** maternal obesity, prebiotic, probiotic, offspring, gut microbiome, transgenerational cycle of obesity

## Abstract

Worldwide, obesity prevalence is rising, severely impairing the health of those affected by increasing their risk for developing non-communicable diseases. The pathophysiology of obesity is complex and caused by a variety of genetic and environmental factors. Recent findings suggest that obesity is partly caused by dysbiosis, an imbalanced gut microbiome. In the context of pregnancy, maternal dysbiosis increases the child’s obesity risk, causing an intergenerational cycle of obesity. Accordingly, interventions modulating the gut microbiome have the potential to interrupt this cycle. This review discusses the potential of pre- and probiotic interventions in modulating maternal obesity associated dysbiosis to limit the child’s obesity risk. The literature search resulted in four animal studies using prebiotics as well as one animal study and six human studies using probiotics. Altogether, prebiotic supplementation in animals successfully decreased the offspring’s obesity risk, while probiotic supplementation in humans failed to show positive impacts in the offspring. However, comparability between studies is limited and considering the complexity of the topic, more studies in this field are required.

## Introduction

With around 1.9 billion adults and over 340 million adolescents and children affected, overweight and obesity, cause 2.8 million deaths every year (1). Overweight and obesity increase the risk for developing non-communicable diseases. As a consequence, health care costs are rising, making obesity one of the biggest health burdens worldwide (1). According to the World Health Organization (WHO) an adult is overweight with a body mass index (BMI) ≥ 25 and obese with a BMI ≥ 30 (1). Genome wide association studies have identified several gene loci associated with obesity. But by accounting for just 2% of the observed BMI variation, these explain only a fraction (2, 3).

Today it is known that several genetic and environmental components affect an individual’s risk of developing obesity and that the obesity risk can be influenced as early as prenatally (4). One factor associated with increased childhood obesity is increased birth weight, which in turn is positively correlated with excessive pre-pregnancy weight (5). With almost 25% of all pregnant women in the U.S. obese, interventions targeting the mother are needed (6). Mechanistically maternal obesity seems to increase the child’s obesity risk over several routes, including elevation of fetal adipocyte quantity, increase in infant energy intake, reduction in infant energy expenditure and epigenetic alterations (2, 7). Epigenetic alterations include hypomethylation of the Zfp423 promoter, a transcription factor needed for adipogenic cell line commitment, and hypermethylation of genes regulating fatty acid oxidation (8, 9).

**FIGURE 1:**
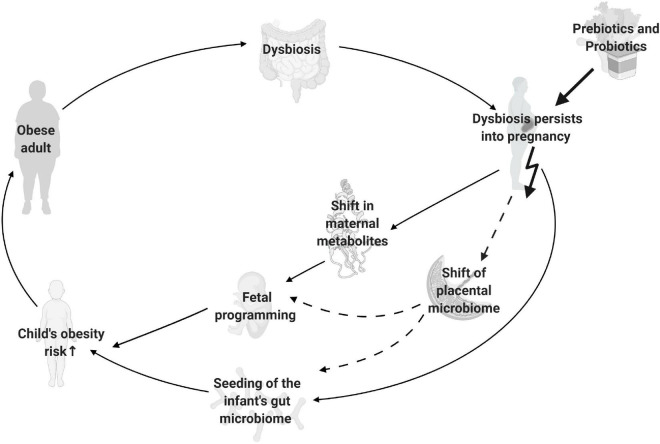
Intergenerational cycle of obesity. Depicted are gut microbiome mediated factors, which increase offspring’s obesity risk in case of maternal obesity. Solid lines represent well established mechanisms, dashed lines represent possible mechanisms with controversial findings. The bold arrow represents the site where prebiotic and probiotic interventions are used to potentially interrupt the cycle. Created with BioRender.com.

One environmental component that gained attention during the past decades is the gut microbiome. Growing evidence suggests that intestinal bacteria significantly affect an adult’s metabolic health and that the composition distinctly differs between individuals with normal weight and obesity (10, 11). Furthermore during pregnancy the maternal gut affects the offspring’s development, metabolic health, and gut microbiome seeding (12, 13). Hence, maternal dysbiosis can increase the offspring’s risk for developing obesity resulting in a transgenerational cycle of obesity (14, 15) (Figure 1). By possibly reversing maternal dysbiosis, interventions such as prebiotics and probiotics have the potential to interrupt this transgenerational cycle (10, 15).

This review aims to compile all existing evidence concerning the effect of prebiotic and probiotic supplementation during obese pregnancy on offspring’s obesity risk, thereby creating an overview of the most promising approaches.

## Background

The following sections discuss the role of the gut microbiome in obesity, with a special focus on how maternal obesity can influence the offspring’s obesity risk and how it can potentially be mitigated using prebiotic and probiotic interventions.

### Gut Microbiome and Its Role in Obesity

With trillions of bacteria, fungi, viruses, protozoa, and archaea the human gut microbiome is an extremely complex system, affecting the hosts state of health (16, 17). Most of the bacteria colonizing the gut belong to one of three main phyla, namely Firmicutes, Bacteroidetes, and Proteobacteria (17). Out of balance (dysbiosis), the microbiome can increase the risk for developing diseases, such as obesity, inflammatory bowel disease and asthma (18). The composition, and therefore its function, is modulated by factors including diet, genetics, environment, and medication (19). Obesity induction upon fecal transplant from an obese mouse into a germ free mouse indicates causality between the microbiome and obesity (20). Regardless, no distinct microbial pattern was found throughout the obese populations. For instance, an increased ratio of Firmicutes/Bacteroidetes was thought to be a hallmark of obesity, in animals and humans alike (19). However, recent studies have shown contradicting results by finding decreased and consistent ratios, questioning the importance of the Firmicutes/Bacteroidetes ratio (17, 19). This might be partly caused by the usage of different methodologies for DNA isolation, amplification region selection, sequencing, and taxa quantification, all of which can lead to over- or underrepresentation of a given strain, causing a deviation from the true sample composition (14, 21, 22). Furthermore, the gut microbiome is affected by external factors such as lifestyle, exercise frequency and ethnicity causing high interindividual variations, further explaining the contradicting results about the Firmicutes/Bacteroidetes ratio (14, 23, 24). Interestingly, Payne et al. (25) demonstrated significant differences in metabolites and their utilization in obese children, despite no numerical difference in the Firmicutes/Bacteroidetes ratio. They conclude that the existing dysbiosis, resulting in overactive metabolic activity, could over time mediate the altered population ratios identified in adults. Thus, despite inconsistencies in the data, certain associations between specific genera/species and BMI exist and will be discussed throughout this review.

The gut microbiome has several functions, including protection against pathogens, synthesis of minerals, and vitamins, bile acid conversion, regulation of inflammation and modification of mucin abundance and gut hormone levels (17, 26, 27). The last includes glucagon- like peptide 1 (GLP-1), glucagon- like peptide 2 (GLP-2), ghrelin, leptin, and peptide YY (PYY), all of which regulate appetite (17). The functions of the gut microbiome are partly mediated by short chain fatty acids (SCFA), the product of plant polysaccharide fermentation by glycoside hydrolase, an enzyme encoded by microbial genes (2). Three main SCFA are produced in the human gut: butyrate, propionate and acetate (28). Amount and ratio of the different SCFA is dependent on the gut microbiome composition and diet (28, 29). In the context of obesity, SCFA exerts two contrasting properties by functioning as an energy source as well as a diverse signaling moiety (17). The latter modulates gut integrity, glucose homeostasis, lipid metabolism, appetite, and immune function (19, 26).

### The Maternal Gut Microbiome During Pregnancy

The shift of the intestinal microbiota during pregnancy is represented by an increased amount of pro-inflammatory Proteobacteria and a decreased diversity (30). Human third trimester microbiota transplantation into mice caused pregnancy associated metabolic changes in the mouse, proving the involvement of the gut microbiome in the body’s metabolism (30). Maternal obesity further modulates the pregnancy gut microbiome represented by a decrease in *Bifidobacterium* spp. and *Bacteroides* spp. levels and an increase in Proteobacteria and Actinobacteria levels (31). These changes can negatively impact the fetal development in various ways. For example, the abundance of Bacteroidetes and *Bifidobacterium* spp. is positively associated with levels of folic acid, one of the most important methyl donors, thus causing epigenetic modifications (31).

### Placental Microbiome

Until recently, the placenta was conceived as sterile during healthy pregnancy, however this dogma started to shift during the last decade (32). Advances in technologies allowed for improved analysis of the placenta, exposing the presence of a low diversity placental microbiota, mainly colonized by Proteobacteria (30, 33). It is proposed that microbes from the gut, vagina and the oral cavity are able to reach the placenta, however exact mechanisms are unknown (30, 34). The placental microbiome could possibly explain the link between maternal obesity and inflammation of the placenta and serve as source for initial intestinal colonization of the fetus (30, 35). Despite increasing evidence in support of a placental microbiome, not all studies confirm this finding, and more research is required to draw a definite conclusion (36). To our knowledge, no study has yet investigated the effect of maternal prebiotic or probiotic supplementation on the placental microbiome.

### Maternal Gut Derived Metabolites and Their Effect on the Fetus

As mentioned above SCFA are metabolites produced by the gut microbiome. SCFA can bind various receptors including G protein coupled receptor 43 (GPR43), which induces extensive downstream signaling (37). One of these downstream effects is expansion of the PYY cell population in the colon causing reduced food intake and prevention of diet induced obesity (38). Furthermore, GRP43 KO mice display impaired beta cell function and a reduction in beta cell mass (39). This presents one way how gut derived metabolites can influence an adult’s metabolic health.

A study showed that maternal SCFA are constantly supplied to the fetus, posing the question whether maternal SCFA can also influence the fetus during development. This question was addressed by a study from Kimura et al. (12). They demonstrated that offspring of non-obese germ-free mice developed an obese phenotype despite being raised by foster mothers, while maternal high fiber supplementation of non-obese mice protected offspring against high-fat diet (HFD) induced obesity. Furthermore, maternal supplementation of propionate in mice consuming a low fiber diet was sufficient to suppress obesity induction in the offspring. Given these results and the knowledge gained from experiments with adult animals they hypothesized that the observed anti-obesogenic effect might be mediated by SCFA. They provided evidence for this hypothesis by showing that SCFA binding to GPR43 causes embryonic differentiation of beta cells and enteroendocrine cells. Hence, maternal SCFA levels influence embryonic insulin levels and embryonic gut satiety hormone secretion, potentially modulating the offspring’s risk for later development of obesity. However, more studies are needed to confirm and further explore these findings.

### Seeding of the Infant’s Gut Microbiome

Maternal microbiome, mode of delivery, early environment and mode of feeding are several relevant factors determining seeding and establishment of the infants gut microbiome (13). The influence of mode of delivery was demonstrated in a recent meta- analysis, showing an increased risk for developing obesity in children born via cesarean section (40). The prevalence of births via cesarean section is higher in obese women (41). One consequence of cesarean sections is a negative alteration of the infant’s gut seeding (2). This alteration is mainly due to the different origin of the intestinal microbiota in cesarean section born infants compared to vaginally borne infants. Namely, intestines of babies born by cesarean section are colonized by bacteria present on the skin and in the environment, while vaginally born babies acquire bacteria present in the maternal vagina and gut (2). If born vaginally, obesity associated changes in the maternal gut microbiome can be transmitted to the infant during birth, altering the abundance of *Bacteroides* spp. and *Staphylococcus* spp. (2). As mentioned, mode of feeding is another relevant factor influencing the seeding of the gut microbiome (13). Breastmilk contains several living bacterial strains such as Streptococcus, Staphylococcus, Serratia, Pseudomonas, Corynebacteria, Ralstonia, Propionibacterium, Sphingomonas, and Bradyrhizobiaceae as well as human milk oligosaccharides, a substance which is not digested by the infant, but promotes growth of beneficial *Bifidobacterium* and *Bacteroides* spp. (42, 43). The positive effects of breastmilk on the gut microbiome may partially explain the findings from a meta-analysis showing that breastmilk is protective against childhood obesity (44). However, breastmilk is an extremely complex substance with many bioactive substances such as hormones and immunoglobulins (43). Additionally, breastfeeding seems to improve the satiety response in infants compared to bottle feeding, thereby further influencing weight regulation (45). Thus, the protective effect of breastfeeding on infant obesity risk is multifactorial, and the extent to which this effect is mediated by the infant gut microbiome remains to be determined.

### Prebiotics and Probiotics

The international scientific association of prebiotics and probiotics (ISAPP) defined prebiotics as “a substrate that is selectively utilized by host microorganisms conferring a health benefit” (46). Other substances than prebiotics can be fermented by the gut microbiome, however, to be classified as a prebiotic the substance must be usable for only a selective group of beneficial microorganisms, allowing for compositional changes of the existing intestinal microbiota (46). Moreover, by reducing luminal pH, prebiotics can inhibit growth of pathogenic bacteria (47). Fermentation of prebiotics produces SCFA, further changing the metabolic milieu, the immune system and thus cytokine expression in the gut (47). For instance, butyrate exerts its anti-inflammatory effect by activating regulatory T cells (47). However, for determination and better understanding of long term effects more studies are needed (47).

Probiotics are “Live microorganisms that, when administered in adequate amounts, confer a health benefit on the host” (48). Hence, probiotics may normalize obesity associated dysbiosis, representing a potential low-cost intervention to fight obesity (26). The majority of probiotics are composed of one or more strains belonging to the genera *Lactobacillus* spp. and/or *Bifidobacterium* spp. (26). The use of different strains in diverse settings showed multiple effects, with certain functions being consistently found, while other properties were strain specific. For instance, administration of 1 × 10^10^ colony forming units *L. rhamnosus* in pregnant women for 4 weeks before expected delivery reduced initial weight gain in children at ages 3, 6, 12, 24, and 48 months, L. acidophilus was bifidogenic, and *L. salivarius* was able to influence gut microbiome properties and the immune system (26, 49). Moreover, strains of *B. animalis* were shown to interact with enterocytes, allowing easier colonization, improved dysbiosis, and protection against infection (26, 34). Functions of the most widely used probiotic strain *Bifidobacterium animalis* subsp. *lactis* BB-12 include immune system modulations, barrier function improvement, reduction of pathogenic growth and maintenance of a healthy microbiome (50). Research suggests a superior efficacy of multi-strain probiotics compared to single strain probiotics in fighting obesity, however the most potent combination of strains is still unknown (26). None of the strains had any known adverse side effects (26, 51). In conclusion, probiotic supplementation changes the intestinal environment, inhibits pathogenic growth and promotes growth of beneficial bacteria, resulting in a healthy gut microbiome (47). However, more research is needed to fully understand their potential in the fight against obesity.

Two additional points must be considered when studying pre- and probiotics. First it is important to note that many factors, especially diet, influence our gut microbiome, making it hard to investigate the effects of gut modulating substance like pre- and probiotics in isolation (52). Secondly the term pre- and probiotics comprises a huge variety of substances all of which have many diverse and complex effects, complicating comparison between different studies which usually administer varying pre- and probiotics.

## Animal and Human Studies Using Prebiotics and Probiotics During Obese Pregnancy

Against the above background, prebiotic, and probiotic interventions represent an interesting option for interrupting the intergenerational cycle of obesity. The following part discusses animal and human studies investigating the effect of prebiotic and probiotic interventions during obese pregnancy on offspring’s risk for developing obesity.

### Method

Publications were searched in the databases PubMed, Cochrane, Scopus, ClinicalTrials.gov, and Google scholar form their inception to February 21, using combinations of the keywords “maternal obesity, prebiotic, probiotic, offspring, gut microbiome, and transgenerational cycle of obesity.” Abstracts were scanned to determine the relevance for this review. To be included, study participants/animals had to be overweight or obese, pregnant, supplemented with a prebiotic or a probiotic, and the health outcomes of the offspring had to be reported. Furthermore, all studies had to be prospective intervention trials. The literature search resulted in four animal studies using prebiotics (53–56) as well as one animal study (57) and six human studies using probiotics (51, 58–62). An overview of all included animal studies is given in Table 1 and provides information about the animal model, performed intervention, animal diet to induce obesity, and main maternal and offspring outcomes. Table 2 provides an overview of all human studies and contains information about study designs, performed intervention including sample size, probiotic, start, and end of the intervention period, frequency of the intervention, maternal BMI as well as the main outcomes.

**TABLE 1:** Overview of all included animal studies, investigating the effect of prebiotic and probiotic supplementation against the background of maternal obesity^a^.

References	Animal model	Intervention	Diet for obesity induction	Maternal groups	Main maternal outcomes	Main offspring outcomes
Guo et al. (57)	C57BL/6J mice	*Bifidobacterium breve*	HFD	HFD	Insulin levels ↓	Weight at day 14 postnatal onward ↓
		*Lactobacillus acidophilus*		HFD + probiotic	Restoration of gut microbiome composition	Adult female pups: glucose and insulin levels ↓
		*Lactobacillus casei*		Control		Restoration of gut microbiome composition on genus level
		*Staphylococcus thermophilus*				
Paul et al. (53)	Sprague-Dawley rats	OFS	HFS	HFS	Gestational weight gain ↓	Weight at day 14 postnatal onward ↓
				HFS + OFS	Gut hormone levels ↑	% body fat↓
				Weight matched	Partial restoration of gut microbiome composition	Fat mass↓
					Altered SCFA levels	Gut hormone levels ↑
					*Bifidobacterium* spp. ↑	*Bifidobacterium* spp. ↑
					*Bacteroides/Prevotella* ↑	*Bacteroides/Prevotella* ↑
					*Methanobrevibacter* spp.↓	
Paul et al. (54)	Sprague-Dawley rats	OFS	HFS	HFS	No maternal outcomes measured	% body fat, fat mass ↓
				HFS + OFS		After weaning: weight of females ↓
				Lean control		AUC glucose levels ↓
						Insulin levels↓
						*Bifidobacterium* spp. ↑
						*Akkermansia muciniphila* ↓
						Risk for developing steatosis↓
Zhang et al. (55)	C57BL6/J mice	Inulin	HFD	HFD	Fasting glucose levels ↓	Birth weight ↓
				HFD + Inulin	Gestational weight gain ↓	Restoration of gut microbiome composition
				Lean control		Butyrate producing bacteria ↑
Maragkoudaki et al. (56)	C57BL6/J mice	PDX	Ob	Ob,	Glucose levels ↓	Weight at month 6 ↓
				Ob + PDX,	Inflammation markers ↓	AUC and peak glucose levels ↓
				Lean control		Protective effect against weight gain in obesogenic environment In males: *Bacteroides* spp. ↑ at weaning

*^a^AUC, Area under the curve; HFD, High fat diet; HFS, High- fat/sucrose diet; Ob, obesogenic diet; OFS, Oligofructose; PDX, Polydextrose; SCFA, Short chain fatty acids.*

**TABLE 2:** Overview of all included human studies, investigating the effect of probiotic supplementation during obese pregnancy^a^.

References	Study design	Intervention| control (sample size at allocation)	Probiotic	Start and end of intervention period given in gestation week	Frequency of intervention	Maternal BMI	Main outcomes (All maternal outcomes, except if otherwise specified)
Lindsay et al. (51)	Double blind RCT	Probiotic capsule | placebo capsule	*Lactobacillus salivarius*	24 until 28	Daily	≥ 30 and < 40	No effect of probiotic intervention
		(83| 82)					
Callaway et al. (58)	Double blind RCT	Probiotic capsule| placebo capsule	*Bifidobacterium animalis* ssp. *lactis*	< 20 until delivery	Daily	≥ 25	Excessive gestational weight gain ↓
		(207| 204)	*Lactobacillus rhamnosus*				Fasting glucose at gestational week 28 ↑
Okesene-Gafa et al. (59)	Double blind RCT	Probiotic capsule| placebo capsule	*Bifidobacterium animals* ssp. *lactis BB12*	12–17 until delivery	Daily	≥ 30	No effect of probiotic intervention
		(115| 115)	*Lactobacillus rhamnosus GG*				
Pellonperä et al. (60)	Double blind RCT	Probiotic capsule| prebiotic capsule	*Bifidobacterium animalis* ssp. *lactis 420*	< 18-until 6 months postpartum	Daily	≥ 25	No effect of probiotic intervention
		(110| 110)	*Lactobacillus rhamnosus HN001*				
Asgharian et al. (61)	Double blind RCT	Probiotic yogurt| conventional yogurt (65| 65)	*Bifidobacterium animals* ssp. *lactis BB12* *Lactobacillus acidophilus La5*	24 until delivery	Daily	≥ 25	Fasting glucose levels after 4 weeks of intervention↓
							2 h OGTT glucose levels at gestational week 28 ↓
							Offspring: Bilirubin levels ↓
Halkjaer et al. (62)	Double blind RCT	Probiotic capsule| placebo capsule (25| 25)	*Bifidobacterium breve*	14–20 until delivery	Twice daily	≥ 30 and < 35	Alpha diversity ↑
			*Bifidobacterium longum*				*Bifidobacterium* spp. levels ↑
			*Bifidobacterium infanti*				*Lactobacillus* spp. levels ↑ Staphylococcus salivarius ↑
			*Lactobacillus acidophilus*				
			*Lactobacillus plantarum*				
			*Lactobacillus paracasei*				
			*Lactobacillus delbrueckii* ssp. *Bulgaricus*				
			*Streptococcus thermophilus*				

*^a^OGTT, oral glucose tolerance test; RCT, randomized control trail.*

## Discussion

The following section discusses the most relevant maternal and offspring outcomes related to offspring’s obesity risk and compares results from animal and human studies. However, it must be considered that numbers of publications are limited, and certain outcomes were not measured in all studies, minimizing the amount of available data. Moreover, with just a single publication, the effect of probiotics in obese pregnant animals is understudied. Likewise, the impact of human prebiotic supplementation in the context of maternal obesity and obesity risk in the child has not been studied to date. Thus, comparability between prebiotic and probiotic interventions is limited, especially since it is unknown to what extent results from animal studies are directly translatable to humans and vice versa. Varying prebiotics and probiotics used, distinct study populations, and differences in data collection further modulated the study results, complicating their comparison. Certain bacterial compositions are thought to be universally associated with health, however, recent publications question, if this is equally valid for all ethnicities (14). Hence, certain links between bacterial strains and health might not hold true for the entire population. Furthermore, the chronology of the publications must be kept in mind since the majority of results from the discussed animal studies were not available during planning of the randomized control trials (RCT). Hence, RCTs are likely based on findings of previous studies, such as prebiotic and probiotic interventions during healthy pregnancies.

### Maternal Outcomes

#### Gestational Weight Gain

In healthy women, excessive gestational weight gain increases the child’s obesity risk by 33–40%, illustrating the importance of appropriate gestational weight gain (63). However, this might not be applicable to women with excessive pre-pregnancy weight. According to a recent meta-analysis the increased obesity risk for offspring of overweight and obese women is mainly caused by excessive pre-pregnancy weight, while excessive gestational weight gain has only a limited additional impact (63). Nevertheless, excessive gestational weight gain is associated with adverse neonatal outcomes besides childhood obesity, therefore reduction of excessive gestational weight gain in obese women is still crucial (64). Five of the RCTs included in this review reported weight gain during pregnancy with a prevalence of excessive gestational weight gain of up to 80% (51, 58, 59, 61, 62). Given the high prevalence of excessive gestational weight gain among obese women, this is not surprising (64). Categorization of excessive gestational weight gain was performed according to the institute of medicine (IOM) guidelines (65). The underlying mechanisms of excessive gestational weight gain are complex, partly mediated by maternal dysbiosis (4). Hence, balancing the intestinal microbiota by administration of prebiotics or probiotics may reduce excessive gestational weight gain. However, only one RCT showed reduction in excessive gestational weight gain by probiotic administration, but no improvement in mean weight gain, complicating the interpretation of these findings (58). At the earliest, probiotic supplementation was provided at gestational week 14 (62), but in some cases only from gestational week 24, which may have been too late to influence gestational weight gain. Furthermore, maternal gut microbiome analysis was only performed in one study (62), hence it is unclear whether all probiotic interventions successfully reached and modified the gut. To get a more profound understanding, further studies sampling the maternal gut microbiome and using varying probiotics, intervention starting points and intervention periods are required. Three out of four animal studies using prebiotics reported weight gain (53, 55, 56), with a successful reduction in two studies (53, 55). Gestational weight gain was not reported in the animal study using probiotics (57). Hence, it is unclear whether prebiotics are more effective than probiotics or if effects observed in animals are not translatable to humans.

#### Maternal Metabolic Health

Glucose, insulin, and gut hormone levels are all linked and frequently dysregulated in obesity. In case of pregnancy, the maternal metabolic health affects the offspring. For instance, high maternal glucose levels are associated with higher childhood adiposity, independent of maternal BMI (66). Hence, reduction of maternal glucose decreases offspring’s risk for developing obesity. Given that the gut microbiome is involved in several metabolic pathways, probiotic and prebiotic supplementation can cause an improvement in maternal metabolic health. For instance by increasing SCFA levels, resulting in improved insulin sensitivity and glucose tolerance, which is mediated by enhanced GLP-1 secretion (47, 67). Furthermore, gut bacteria are involved in the conversion of primary bile acids into their secondary form, allowing bile acids to bind the TGR5 receptor, further stimulating GLP-1 secretion (10). Additional to GLP-1 secretion stimulation TRG5 activation by bile acids induces the TGR5- cAMP pathway in brown adipose tissue, whereby deiodinase 2 is upregulated, eventually increasing mitochondrial oxidative phosphorylation and energy expenditure (68). TGR5 induced increase in energy expenditure and TGR5 dependent stimulation of GLP-1 can potentially reduce body weight. Obesity associated changes in the gut microbiome can trigger an inflammatory response, causing alterations of the closely linked metabolic system (19). For instance, an increase in inflammation leads to modification of insulin signaling cascades, potentially contributing to insulin resistance (69). A meta-analysis in healthy pregnant women showed successful reduction of glucose levels with probiotic administration (70). However, results of the here discussed RCTs are inconclusive, mostly not showing an impact of probiotic supplementation (51, 58–62). Similar, where measured, supplementation did not affect insulin levels (51, 60), possibly as a result of the selection of probiotic strains. Likewise, probiotic administration in obese mice did not reduce plasma glucose levels (57). However, insulin concentrations were decreased after probiotic administration in obese mice, which correlated with the abundance of *Bacteroides* spp. (57). Interestingly, another study by Castro-Rodríguez et al. in obese pregnant rats found a reduction in fasting glucose levels as well as mitigation of insulin resistance after supplementation with the probiotic Leuconostoc SD23 (71). Rats were supplemented from 1 month before pregnancy up until lactation. This study was not included in the review since they did not perform any examinations on the offspring. The study by Castro-Rodríguez et al. shows that a longer duration or the use of a different probiotic may improve the potential of probiotics. Administration of prebiotics in obese pregnant rodents reduced maternal glucose levels in two out of three studies and was not reported in the fourth (53–56). Only one study using prebiotics measured insulin and gut hormone concentrations. While insulin concentrations remained unchanged, gut hormone levels were increased upon supplementation (53). Possible influences of the intestinal bacteria on gut hormone levels will further be discussed below. Certain genera and species, such as *Bifidobacterium* spp. and *Akkermansia muciniphila*, have been associated with insulin sensitivity and glucose tolerance (2, 19), however establishment of a constant pattern between any given strain and insulin or glucose levels was not possible in this review as most studies used multiple strains in different combinations. Comparison between studies was complicated by several factors, including reporting of different bacteria, use of a variety of methods and time points, and an almost complete lack of microbiome analysis in the RCTs. Hence, it remains unexplained whether prebiotics and probiotics modulate the gut in a comparable manner. Since the majority of the used probiotics consisted of just two strains, supplementation with probiotics potentially exhibit a narrower function compared to prebiotics, that provide nourishment for a variety of strains (26, 46). This might explain why probiotic interventions in humans failed to consistently improve maternal metabolic health, while use of prebiotics in mice was more effective. However, it is yet to be determined whether this is translatable to humans.

#### Maternal Microbiome

The pregnancy induced shift in the gut microbiome is more pronounced in case of maternal obesity, revealing persistence of obesity associated dysbiosis into pregnancy (72). For instance, maternal obesity amplifies the decrease in alpha diversity, a measure of microbial diversity, during the last trimester (14, 31). Probiotic supplementation successfully increased alpha diversity in the one RCT which examined the maternal gut microbiota in obese pregnancy (62). On the other hand, in an animal study, alpha diversity was significantly decreased in probiotic treated and lean control dams compared to obese, non-supplemented dams (57). Given that weight gain and diet are two distinct factors influencing the gut microbiome (73), the seemingly controversial higher alpha diversity in obese dams is possibly caused by a high- fat diet in the animal study (57). However, comparison of several studies revealed no constant pattern of alpha diversity and high fat feeding (73), depicting the complexity and the influence of unknown factors. On genus level, probiotic and prebiotic interventions modified the maternal gut microbiome composition, confirming the feasibility of the interventions in animals and humans alike (53, 57, 62). However, the findings in humans are only based on one RCT by Halkjaer et al. (62). Whether results from this study are representative for all studies remains unclear, especially since they differ in various aspects. For instance, whereas the study by Halkjaer et al. used a probiotic consisting of eight strains, all the others used probiotics consisting of two strains at most. Given the proposed increased functionality of multi-strain probiotics, this might affect their efficacy (26). Furthermore, potency and function depend on the strains and combinations used. Hence, the available data does not allow to determine whether all probiotics successfully modulated the intestinal microbiota and if so, in which direction. Accordingly, lacking effects in maternal and infant outcomes are not necessarily caused by a lacking efficacy of probiotics in general but rather illustrate the need for other strains and combinations.

### Offspring’s Outcomes

#### Birth Weight

Elevated birth weight is associated with a higher incidence of childhood obesity which in turn predicts a person’s risk for being obese in adulthood (15). Hence, birth weight is an important predictor determining obesity risk. Prebiotic administration in rodents decreased pups birth weight in only one study (55). However, in three additional studies pups weight was significantly reduced at day 14 (53), at 6 months (56), or after weaning (only in females) (54). Furthermore, one study noted a reduction in fat mass in pups of prebiotic treated dams, despite unchanged birth weight (54). Similarly, in the one available study, maternal probiotic administration lowered pups weight at day 14, which persisted into adulthood (57). Interestingly, no RCT showed an effect of maternal probiotic supplementation on birth weight (51, 58–62). However, considering the results from rodents, measurements of infants’ body composition as well as longer follow up periods might be necessary to reveal an effect of maternal supplementation in humans.

#### Offspring’s Metabolic Health

As discussed above, composition of the intestinal microbiota affects the hosts metabolic health. Hence, alterations in infants gut microbiome seeding, caused by factors such as maternal dysbiosis, increase the child’s risk for metabolic dysregulations (74). These dysregulations can partly be analyzed by measuring insulin, glucose, and gut hormone levels. Insulin levels were only reported in three animal studies using prebiotics (53–55), with two showing an improvement in insulin sensitivity upon maternal supplementation (54, 55). On the other hand, prebiotic administration improved glucose control in all four studies (53–56). Although probiotic supplementation in mice had no effect on young pups, female metabolic profiles improved when reaching adulthood (57). The one RCT measuring offspring’s glucose levels, showed no effect of probiotic administration (51). Given that the RCTs differed in probiotic composition and complexity, this result might not be representative for all studies. The mentioned study used a single strain probiotic (51), hence modulation of the intestinal microbiota might be less broad compared to a multi- strain probiotic. Thus, those results may lead to an underestimation of the effect of general probiotic interventions, as a more profound modulation of the gut microbiome would be possible with a multi- strain probiotic. Similar to multi- strain probiotics, prebiotics elicit a broader modification of the gut microbiome, likely explaining the differences observed in the results. Furthermore, based on the results from the animal studies, longer follow up times or analyses stratified for sex may be required rather than measurements at birth only.

The one animal study that measured gut hormone levels demonstrated an increase in pups of prebiotic supplemented dams (53). As discussed in the first part, prebiotics can mediate this effect by different mechanisms including improved differentiation of enteroendocrine cells (12). Moreover, given the involvement of gut bacteria in hormone production, changes in seeding and establishment of the intestinal microbiota can alter offspring’s hormone levels (27). For instance, butyrate is known to increase GLP-1 levels, hence the reported increase in butyrate producing bacteria after prebiotic supplementation by Zhang et al. could indicate an increase in GLP-1 (55). However, this remains speculative as most studies did not measure hormone concentrations and thus more studies are required to link microbiome compositions to hormone profiles.

#### Infant Microbiome

Despite some association between the gut microbiome composition in infants and obesity, results are often conflicting, not least because of the sheer abundance of gut microbes. Comparison between studies is further complicated by the use of various technologies, measurement of different genera and species as well as intervention variability. Despite this variability, maternal prebiotic supplementation induced a shift of microbial composition in the offspring in all studies (53–56). For instance, administration of the prebiotic oligofructose (OFS) resulted in an increase of *Bifidobacterium* spp. in the offspring’s gut microbiome (53, 54). This bifidogenic effect of OFS is well established. Among other things, *Bifidobacterium* spp. produce SCFA and lactate, enabling stimulation of the offspring’s immune system and acidification of the intestine. This results in the offspring’s protection against certain diseases and is associated with better health (34, 53). Furthermore, low levels of *Bifidobacterium* spp. are associated with inflammation, insulin resistance, obesity and type 2 diabetes (2). *Bifidobacterium breve*, a species where a low abundance is linked to obesity, was increased in offspring of inulin supplemented dams (55). Furthermore, maternal obesity increased abundance of Proteobacteria in pups (55). This increase is associated with a delayed intestinal maturation (4, 55), which influences immune system development, causing an elevated risk for acquiring immunological diseases, such as asthma and allergies (4, 34, 75). Inulin supplementation decreased Proteobacteria levels, demonstrating its ability to modulate the intestinal microbiota, improving pups health (55). Administration of Polydextrose (PDX) increased male pup’s levels of *Bacteroides* spp., a genus positively associated with weight loss (56). A probiotic consisting of four strains successfully shifted the microbial composition in pups, confirming feasibility of the intervention (57). None of the existing human studies performed analysis of the infant gut microbiome (51, 58–62).

## Conclusion and Outlook

Without dispute the gut microbiome immensely influences our wellbeing. During pregnancy, maternal intestinal bacteria and their metabolites impact the fetal development and influence the child’s risk for numerous diseases including obesity. Probiotic interventions have proven to be safe and feasible in humans, however, their efficacy in reducing offspring’s obesity risk is controversial. Prebiotic interventions in animals show promising results; however, transferability to the human system is yet to be confirmed. Further large- scale studies that also consider confounding factors such as mode of feeding, antibiotic use, or cesarean section are required to fully assess the potential benefits of prebiotic and probiotic supplementation during obese pregnancy. Implementation of microbiome analyses in future studies will allow to expand our knowledge of the intestinal microbiota, allowing development of more potent and better tailored prebiotic and probiotic interventions. Furthermore, longer term follow-up will be crucial to investigate to what extent observed effects at birth persist and effects that only occur at a later point.

## Author Contributions

EW was responsible for writing the first draft of the manuscript. IH-A revised the manuscript. Both authors have read and approved the final manuscript.

## Conflict of Interest

The authors declare that the research was conducted in the absence of any commercial or financial relationships that could be construed as a potential conflict of interest.

## Publisher’s Note

All claims expressed in this article are solely those of the authors and do not necessarily represent those of their affiliated organizations, or those of the publisher, the editors and the reviewers. Any product that may be evaluated in this article, or claim that may be made by its manufacturer, is not guaranteed or endorsed by the publisher.

## Abbreviations

GLP-1: glucagon like peptide 1
GLP-2: glucagon like peptide 2
GPR43: G protein coupled receptor 43
HFD: high fat diet
IOM: institute of medicine
OFS: oligofructose
PDX: Polydextrose
PYY: Peptide YY
RCT: randomized control trail
SCFA: short chain fatty acids.
